# Impact of Proton Beam Irradiation on the Growth and Biochemical Indexes of Barley (*Hordeum vulgare* L.) Seedlings Grown under Salt Stress

**DOI:** 10.3390/plants9091234

**Published:** 2020-09-18

**Authors:** Lacramioara Oprica, Marius-Nicusor Grigore, Iulia Caraciuc, Daniela Gherghel, Cosmin-Teodor Mihai, Gabriela Vochita

**Affiliations:** 1 Faculty of Biology, Alexandru Ioan Cuza University, 20A Carol I Bd., 700506 Iasi, Romania; iasilacra@yahoo.com; 2 National Institute for Research and Development in Electrical Engineering ICPE-CA, 313 Splaiul Unirii, District 3, 030138 Bucharest, Romania; iulia.caraciuc@icpe-ca.ro; 3 Institute of Biological Research—Branch of NIRDBS, 47 Lascar Catargi Street, 700107 Iasi, Romania; cosmin-teodor.mihai@umfiasi.ro (C.-T.M.); gabrielacapraru@yahoo.com (G.V.); 4 Advanced Center for Research and Development in Experimental Medicine (CEMEX), Gr.T.Popa Medicine and Pharmacy University of Iasi, 9-13. M. Kogalniceanu, 700115 Iasi, Romania

**Keywords:** beam proton radiation, saline stress, antioxidant protection systems, barley

## Abstract

The present paper examines the effects of salt stress on the growth, pigments, lipid peroxidation and antioxidant ability of barley (*Hordeum vulgare* L.) seedlings raised from proton beam irradiated caryopses. In order to assess the effects of radiation on the early stages of plant growth and analyze its possible influence on the alleviation of salinity, 3 and 5 Gy doses were used on dried barley seeds and germination occurred in the presence/absence of NaCl (100 mM and 200 mM). After treatment, photosynthetic pigments increased in the 5 Gy variant, which registered a higher value than the control. Among the antioxidant enzymes studied (SOD, CAT, and POD) only CAT activity increased in proton beam irradiated seeds germinated under salinity conditions, which indicates the activation of antioxidant defense. The malondialdehyde (MDA) content declined with the increase of irradiation doses on seeds germinated at 200 mM NaCl. On the other hand, the concentration of 200 mM NaCl applied alone or combined with radiation revealed an increase in soluble protein content. The growth rate suggests that 3 Gy proton beam irradiation of barley seeds can alleviate the harmful effects of 100 mM NaCl salinity, given that seedlings’ growth rate increased by 1.95% compared to the control.

## 1. Introduction

Barley (*Hordeum vulgare* L.), which belongs to the Poaceae family, is the fourth most important cereal crop in the world after wheat, corn, and rice and is almost in the top ten crop plants in the world. Barley is used to produce starch, either for food or for the chemical industry. Although in the past barley was mainly consumed by people, today it is usually used to feed livestock and to produce malt, and only in small quantities for direct human consumption. In addition, barley has medicinal applications in many diseases, such as anti-cough, bladder inflammation, blood sugar levels, cholera, dermatitis, diabetes [1,2].

Barley is a salt-tolerant species that presents a greater degree of salt tolerance compared to rice (*Oryza sativa*) and wheat (*Triticum aestivum*). Barley is most sensitive to salinity at the time of germination and in the early seedlings stage, but it becomes more tolerant as it grows. Saline sensitivity in the seedling stage has been attributed mainly to the ionic effects and lesser to the osmotic action [3,4]. Some studies revealed a wide interspecific variability for salt tolerance in cereals during germination, and the response to salt depends on the species, the variety, the salt concentration of the field, and the plant development stage [5]. Generally, salinity is known to be one of the most common environmental stresses affecting physiological and biochemical processes.

In order to alleviate salinity stress, seeds, seedlings, or plants are exposed to several chemical, biological, and physical treatments. In this way, a series of molecular and physiological mechanisms are activated, which enables the seed or the plant to respond faster and/or stronger after exposure to salinity [6].

Various strategies have been used to improve crop growth in saline conditions, including the production of salt-tolerant genotypes. These may include the use of the plant growth regulators—PGR [7,8], the phytohormones [9,10], or plant growth-promoting rhizobacteria—PGPR [11,12] in order to diminish or eliminate the negative effects of salinity and to obtain the plants that are able to manage salinity stress. In addition, numerous studies of gamma radiation and salinity effects, applied separately or simultaneously, on mono- or dicotyledonous species, such as: *Oryza sativa* [13,14], *Medicago sativa* [15], *Ambrosia maritime* [16], *Vigna sinensis* [17], *Osmanthus fragrans* [18], *Moringa oleifera* [19], have been performed. According to Macovei et al. [13], gamma irradiation treatment can enhance the resistance of some plants to abiotic stress conditions, such as salt and drought, by intensification of antioxidant enzymes activity and activation of Transcription-Coupled Nucleotide Excision Repair (TC-NER) pathway. However, there is little data on the influence of proton beam irradiation on plant salinity tolerance and even less on that of barley.

Some studies mention that the biological effects of radiation on plants are strongly influenced by a variety of factors related to (i) plant particularities such as: the species and cultivar, the growth stage, the tissue architecture, and hereditary material, and (ii) radiation characteristics, such as intensity, quality, dose-rate, and the duration of exposure [20].

Mutation breeding is an alternative method used to improve agronomic traits, such as stress resistance, physical parameters of grain, and nutritional value. It is known that ionizing radiation helps overcome the limitations of the chemical treatment so that new mutations can be generated in a short period [21,22,23]. There are two forms of ionizing radiation that transfer energy into a substance: non-particulate (gamma and X rays) and particulate (alpha and beta particles, neutrons, and protons). Recent studies revealed that, in barley, ion beams including protons have an important role in mutation breeding, given that positively charged ions are accelerated at a high speed (approximately 20–80% of the speed of light) and constitute the high linear transfer radiation (LET) used for irradiation of target cells [24]. In contrast to low linear energy transfer radiation (e.g., gamma and X-rays), which represents the application of a high amount of energy to a small area, the ion beam provides high LET when aligned along the direction of the beam. The biological effects on plants are bigger in case of higher LET radiation than low LET radiation. Taking into account the structure of the cereal grain, the ion beam can easily penetrate the coat of the caryopses and reach the meristematic tissue of the embryo with a high irradiation efficiency [25].

Some studies [26,27] focus on the use of proton irradiation used to induce genetic variability in crop plants in order to create new varieties with production performance and high tolerance to various stressors (salinity, herbicides, etc.), which are beneficial to farmers.

The biological response to radiations mainly depends on the type of radiation, type of interaction, and also the way energy is deposited in tissues [28,29].

In this paper we dealt with irradiation and salt treatments as abiotic stress factors; therefore, we intended to test the effects of a multiple stress on seedlings of *Hordeum vulgare*. While salt stress solely has a long history in plant physiology and biochemistry, combining it with other types of stress is rather a novel approach; within these other factors, proton beam irradiation has been rarely reported to be used as an additional stressful factor on plants. On the other hand, proton beam has emerged as a novel mutagen in mutation breeding of crop plants. Mutation breeding using ionizing radiation has been used as a useful tool for creating genetic variability and crop improvement. Despite our paper is not focused on mutations in investigated species, it is useful, however, to test some biochemical indices in *Hordeum* sp. under salt stress conditions and irradiation treatment in order to achieve a good picture of plant responses. It has been reported that proton beam can be used in creating novel variability that will be beneficial to the farmers; as *Hordeum* sp. is an important crop, with a certain degree of salt tolerance, it would be beneficial to have some answers regarding the biochemical adaptive profile of this species under a scenario of aridization, salinization, and climate change.

In this context, the present study was designed to explore the physiological and biochemical responses of *Hordeum vulgare* seedling to salt stress (100 and 200 mM NaCl) and to determine how proton beam irradiation pretreatment of seeds (3 and 5 Gy) improves barley tolerance by modulating antioxidant activity and reactive oxygen species (ROS) balance.

## 2. Materials and Methods

### 2.1. Experimental Design of Plant Material and Growing Conditions

Barley (*Hordeum vulgare ssp. vulgare*) seeds were kindly provided by the Agricultural Research and Development Station, Secuieni Neamt, Romania. The study of barley seedling tolerance to salinity and proton beam irradiation pretreatment was conducted under laboratory conditions, based on completely randomized design with three replications.

#### 2.1.1. Proton Beam Irradiation

The monolayer of dry barley caryopses was placed in Petri dishes and exposed to a proton beam accelerated to 150 MeV with a dose of 0, 3, and 5 Gy at room temperature. Irradiation was performed at the protons linear accelerator facility in the phasotron Dzhelepov Laboratory of Nuclear Problems from JINR (Dubna). The beam energy in the cabin was determined by the range of the beam in water (R = 200 mm of water), and the average LET, dE/dx = 0.539 keV/µm, where dE represents energy loss and dx is the increment of path length.

#### 2.1.2. Growth Conditions and Salinity Treatment

The grains used in the study were healthy, smooth, and intact and were of similar size and color. For each variant, twenty-five seeds were disinfected with 5% sodium hypochlorite for 15 min and then thoroughly washed with sterile distilled water. For germination, the caryopses were placed in Petri dishes at 25 °C in the dark for 72 h and then were transferred into a growth chamber at 23 °C under photoperiod conditions—16/8 h (light/dark). Afterward, treatments started by daily addition of 2 mL of salt solutions (or distilled water for the control and single irradiated seeds). Saline stress was replicated by two concentrations of NaCl (100 mM, 200 mM). This study consisted of eight treatments with saline and proton irradiation applied on barley caryopses (alone or combined) as follows: 3 Gy, 5 Gy, 100 mM NaCl, 200 mM NaCl, 3 Gy + 100 mM NaCl, 3 Gy + 200 mM NaCl, 5 Gy + 100 mM NaCl, 5 Gy + 200 mM NaCl. The variant without application of treatments (0 Gy and 0 mM NaCl) was used as control. Physiological and biochemical analyses were conducted at 16-days-old seedlings; five different individuals from each treatment were selected to measure all the analyzed parameters.

### 2.2. Photosynthetic Pigments Analysis

The photosynthetic pigments viz. chlorophyll *a*, chlorophyll *b,* and carotenoids from 16-days-old barley seedlings (10 mg) were extracted with 80% acetone and quantified following Reference [30]. Optical density of supernatant was measured with UV-visible spectrophotometer at 663 nm (chlorophyll *a*), 646 nm (chlorophyll *b*), and 470 nm (xanthophyll and carotenoids). The amount of pigments was evaluated and expressed in mg g^−1^ fresh weight (FW) using the following equations, in agreement as stated by Lichtenthaler [30]:

Chlorophyll *a* (Chl *a*) = (12.21 × A663 − 2.81 × A645)

Chlorophyll *b* (Chl *b*) = (20.13 × A645 − 5.03 × A663)

Carotenoids = [(1000 × A470 − 3.27 × Chl a − 104 × Chl b)/227]

### 2.3. Oxidative Stress Indexes

*Malondialdehyde (MDA)* as an indicator of lipid peroxidation products was quantified in fresh barley leaves according to the method described in Reference [31]. Thus, 0.20 g of samples were collected and homogenized thoroughly in 10% trichloracetic acid (TCA), followed by centrifugation for 10 min at 400 rpm/min. One milliliter from the supernatant sample was mixed with 2 mL of 0.5% thiobarbituric acid (TBA) solution (in 10% TCA). The mixture was kept at 95 °C for 60 min and cooled at room temperature, then centrifuged at 12,000 rpm for 10 min to remove the interfering substances. Absorbance was recorded at 532 nm using UV–VIS spectrophotometer (Model Pharma Spec UV-1700 Shimadzu, Kioto, Japan).

### 2.4. Antioxidant Defense System

#### 2.4.1. Non-Enzymatic Constituents

##### Total Polyphenols Content

The total content of polyphenols was established using the modified Folin-Ciocalteu method [32]. The absorbance of the blue-colored solution obtained was evaluated at 765 nm after two hours, using the distilled water as a blank. The quantity of the total polyphenolic content was expressed as milligram equivalents of gallic acid/gram of dry weight (mg GAE g^−1^ DW) (R^2^ = 0.99). For each sample, three measurements were taken and the averages were calculated.

##### Flavonoids Content

The content of flavonoids was assessed by spectrophotometric method [33]. The absorbance of the pink-colored solution obtained after reactions was estimated at 510 nm compared to the blank (distilled water). Flavonoids amount was expressed as mg catechin equivalent (mg CE g^−1^ DW) (R^2^ = 0.98). The average of the three repetitions for each sample was established.

#### 2.4.2. Antioxidant Enzymes

##### Preparation of Enzyme Extracts

In order to obtain the crude enzyme extract, 0.30 g of fresh 16-days-old seedlings of each sample was homogenized with 3 mL 0.1 M sodium phosphate buffer solution (pH = 7.5). Homogenates were centrifuged at 15,000 rpm/min at 4 °C for 15 min. Supernatants containing crude enzymes were analyzed for activities of superoxide dismutase SOD, CAT, and POD.

##### SOD Activity

SOD activity was evaluated by monitoring its ability to inhibit the reduction of nitro blue tetrazolium (NBT) by the superoxide radicals resulted via reoxidation of photochemically reduced riboflavin [34]. The reaction mixture included 1.5 mL phosphate buffer (50 mM, pH 7.8), 0.5 mL 0.1 mM EDTA, 0.5 mL 130 mM methionine, 0.5 mL 0.5 mM NBT, 0.5 mL 0.02 mM riboflavin, and 0.05 mL enzyme extract with a final volume of 3 mL. Riboflavin was added last, and the samples were illuminated by placing the tubes below a light source of 215 W fluorescent lamps for exactly 5 min. One unit of SOD activity is described as the quantity of enzyme necessary to determine 50% inhibition of the reduction of NBT recorded at 560 nm.

##### POD Activity

POD activity was spectrophotometrically established by measuring the color intensity generated by the oxidation of o-dianisidine (3, 30-dimethoxybenzidine) with H_2_O_2_ in the presence of peroxidase at 460 nm, using the method described by Ranieri et al. [35]. Initiation of the reaction was assured by addition of 50 µL extract to the reaction mixture represented by 20 mM phosphate buffer (pH 5.0), 1 mM o-dianisidine, and 3 mM H_2_O_2_. Specific activity of POD was expressed as the amount of enzyme that produced a change of 1.0 absorbance per min, expressed as units (mM of oxidized dianisidine per min) per mg protein.

##### CAT Activity

CAT activity was determined by Sinha’s assay with minor adjustments [36]. The reaction mixture consisted of 0.4 mL phosphate buffer (0.01 M, pH 7.0), 0.5 mL hydrogen peroxide (0.16 M), and 0.1 mL enzyme extract reaching a final volume of 3.0 mL. About 2 mL dichromate acetic acid reagent was added in 1 mL of reaction mixture, boiled for 10 min, and then cooled. After stopping the catalase action, the amount of unmodified hydrogen peroxide, which reduces potassium dichromate to chromium acetate in an acid medium, was recorded at 570 nm. Therefore, CAT activity is reported as the amount of enzyme needed to reduce 1 µmol of H_2_O_2_ per min.

The activity of all antioxidant enzymes (SOD, POD, and CAT) was expressed as unit per mg proteins (U mg^−1^ protein).

The soluble protein content was assessed by Bradford’s method (1976) [37] using bovine serum albumin as standard. This method consists of the binding of Coomassie Brilliant Blue G-250 to the aromatic amino acid radicals and the measurement of extinction at 595 nm. The results are expressed in mg protein per g fresh weight (FW).

To compare the sensitivity of each parameter, the increase/decrease rates were established by the following equation: (1 − *x*/*y*) × 100, where *y* is the average value detected in the control and *x* is one of each treated sample.

**Statistical analysis.** All experiments were performed using three independent repetitions and the results were expressed as the mean values ± standard errors (SE). The statistical significance of the differences between treated samples and control ones was assessed by means of the Student’s *t* test. The differences were considered as significant at levels of *p* < 0.05 and were represented by statistical lettering. The increase/decrease (−/+) rates were calculated based on the equation: (1 − x/y) × 100, where y is the average value detected in the control and x is one of each treated samples.

## 3. Results and Discussions

### 3.1. Tolerance of Proton Beam Irradiated Barley Seedlings to Salinity Stress

**Figure 1:**
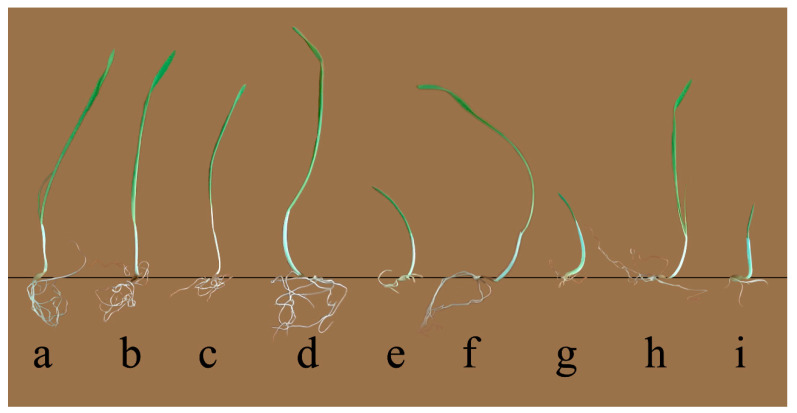
The phenotypic appearance of non-irradiated (0 Gy) and irradiated (3 Gy and 5 Gy) barley seedling grown with (100 and 200 NaCl) and without salinity: (**a**) Control, (**b**) 3 Gy, (**c**) 5 Gy, (**d**) 100 mM NaCl, (**e**) 200 mM NaCl, (**f**) 3 Gy + 100 mM NaCl, (**g**) 3 Gy + 200 mM NaCl, (**h**) 5 Gy + 100 mM NaCl, (**i**) 5 Gy + 200 mM NaCl.

The length of seedlings elongation showed declining tendency with each increasing doses of proton beam radiation as well as salinity concentrations after single treatment application. The results showed that the lowest beam irradiation dose (3 Gy) resulted in a 1.38% decrease in the length of barley seedlings, while in the case of the 5 Gy dose the decrease was 5.10% (Table 1 and Figure 1). In addition, the application of a 100 mM salinity concentrate resulted in a slight increase (5.93%) of seedlings length and a significant decrease (58.62%) in the growth rate at the maximum concentration of NaCl (200 mM). In the case of irradiation pretreatment, after 100 mM NaCl application, the seedlings length at 3 Gy increased by 1.93% and decreased by 4.21% after 5 Gy proton beam irradiation. On the other hand, the results of germination in presence of 200 mM NaCl indicated a significant decrease of barley growth by 72.90% (*p* < 0.001) and 73.93% (*p* < 0.001) respectively, at 3 Gy and 5 Gy proton beam irradiation, respectively. This shows that 3 Gy proton beam irradiation can alleviate the harmful effect of 100 mM NaCl salinity, as demonstrated by the significant growth rates of barley seedlings.

Generally, low doses of irradiation stimulate germination and seedlings growth, while high doses inhibit seeds growth, while the sensitivity of each applied dose varies depending on the type of radiation and the tested species. For example, Wi et al. [38] obtained a slight stimulation of the growth of *Arabidopsis* seedlings when the seeds were exposed to 1 or 2 Gy, while a 50 Gy dose had an inhibitory effect. Singh and Datta [39] showed that 10–100 Gy doses improved the germination potential of wheat seeds. In addition, gamma radiations represent one of the most powerful agents that can alter the physiological and biochemical properties of plants in correlation with the absorbed doses, and higher doses have an inhibitory effect on plants by generation of free radicals with repercussions on physiological, morphological, and anatomical processes depending on the level of irradiation [40,41]. The dry and water-imbibed *Arabidopsis* seeds irradiated with 2.6 MeV H^+^ and 6.5 MeV H^+^ have shown a rapid reduction of germination and survival rate, with water-soaked seeds having a higher sensitivity to irradiation compared to dry ones [42]. Furthermore, the proton beam pretreatment of rice seeds (0, 50, 100, 200, 300, 400, 500, 600, 700, and 800 Gy) induced a significant reduction in the rate of germination (over 30%) at doses higher than 300 Gy, and an increase in the height roots and shoots at 50 and 100 Gy, 14 days after irradiation [43]. Recent studies [44] have revealed a linear decrease in germination rate after the application of increasing doses of the proton beam, ranging from 0–500 Gy, upon Indian rice variety (*Oryza sativa* L.). Similar results were obtained by Bae et al. [45] indicating a decrease in the germination rate of tobacco and rice seeds as the dose proton beam and the irradiation time increased, which suggests that this type of irradiation can be used as a mutagenic agent for obtaining new useful varieties. For example, Gonzalez et al. [46] state that they obtained the first rice cultivar with improved productive potential and tolerance to salinity by proton irradiation of rice seeds belonging to variety J-104. Moreover, Im et al. [47] using 57-MeV proton beam treatment (50–400 Gy) obtained an increase of germination rate but a strong reduction of survival rates in soybean plants. They mentioned that optimal doses for breeding mutation are between 250 and 300 Gy. Regarding the salinity stress effects, growth inhibition is a common response to NaCl and depends on tolerance of the plants/seedling as well as the salt concentration [48].

### 3.2. Effect of Salinity and Proton Irradiation on Photosynthetic Pigments Content of Barley Seedlings

Photosynthetic pigments such as chlorophyll a (Chl *a*), chlorophyll *b* (Chl *b*)*,* and carotenoids were affected in the sense of decreasing them by the applied treatments (Table 2). Regarding the content of Chl *a,* it was observed that was higher than the control, only at 5 Gy. Under salt stress (100 mM and 200 mM NaCl), the pigments content in barley seedlings diminished as salinity increased. Our results are similar to Baek et al. [49], which showed lower chlorophylls content and a decrease in the effective quantum yield of photosystem 2 in the NaCl-treated rice seedlings.

The seeds irradiated with 3 Gy, grown in 100 mM and 200 mM NaCl salinity conditions, revealed a significant decrease in Chl *a* (*p* < 0.01). In addition, a significant decrease was observed in case of Chl *b* at 200 mM NaCl and 3 Gy + 100 mM NaCl (*p* < 0.05) while at carotenoids at 3 Gy + 200 mM NaCl (*p* < 0.01).

The highest decrease in Chl *a* content in barley seedlings, as a response to all types of stress, simple or combined, was at 3 Gy + 100 mM NaCl variant, which was 2.33 times lower compared to the control. A similar pattern to the Chl *a* was observed in the content of Chl *b* and carotenoids in all analyzed samples. Malanga et al. [50] stated that the decrease of the total Chl content could be the consequence of the alteration of the photosynthetic capacity of chloroplasts. Our results are in accordance with those reported by References [51,52] in which total Chl contents decreased with the increase of NaCl concentrations. Several studies have shown that low doses of gamma rays applied on seeds stimulate germination, plant growth, and synthesis of photosynthetic pigments [53]. However, Macovei et al. [13] showed that single gamma irradiation of seeds (25 to 200 Gy) induced significant decrease in chlorophyll content in 20-day-old rice plantlets, which contained a higher level of Chl *b*. On the other hand, the low dose rate irradiated seeds (25 Gy) grown in the presence of 100 mM NaCl revealed a significant increase in Chl *a* and *b*, and a slight difference was recorded after high dose rate (100 and 200 Gy) treatments.

Decrease of chlorophyll content is an important indicator of the senescence foliar process and further a significant marker of physiological reactions in response to xenobiotic stress [54]. Kalimullah et al. [55] mentioned that ^1^H heavy ion radiation can inhibit chlorophyll biosynthesis and, similarly, gamma rays and proton beam irradiation alter photosynthetic pigments, leading to alteration of the photosynthesis process [56].

Compare to the pigments Chl *a* and Chl *b*, the carotenoid content showed the same decreasing tendency, compared to the control, when applying simple and combined treatments of stress and radiation. Carotenoid pigments, component of the plant photosynthetic apparatus and non-enzymatic antioxidants that maintain homeostasis in the plant, can protect cell membranes from the effect of oxidative stress caused by many agents, including salinity or radiations. The carotenoids content in 3 Gy and 5 Gy samples decreased by 33.50% and 9.10%, respectively, compared to the control. The 100 to 200 mM NaCl treatments induced a reduction of the carotenoids level in the barley seedlings by 22.64% and 48.22%, respectively. Consistent with our results, Reference [57] reported that salinity causes a decrease in the content of carotenoid pigments with a negative effect on the process of photosynthesis and the mechanisms of defense against oxidative stress.

### 3.3. Effects of Proton Irradiation on ROS Scavenging Systems of Barley Seedling in Response to Salt Stress

In order to determine the antioxidant responses of barley irradiated seeds (3 and 5 Gy) germinated under saline stress conditions (100 mM and 200 mM NaCl), the enzymatic activity of SOD, POD, and CAT in 16-days-old barley seedlings were measured.

**Figure 2:**
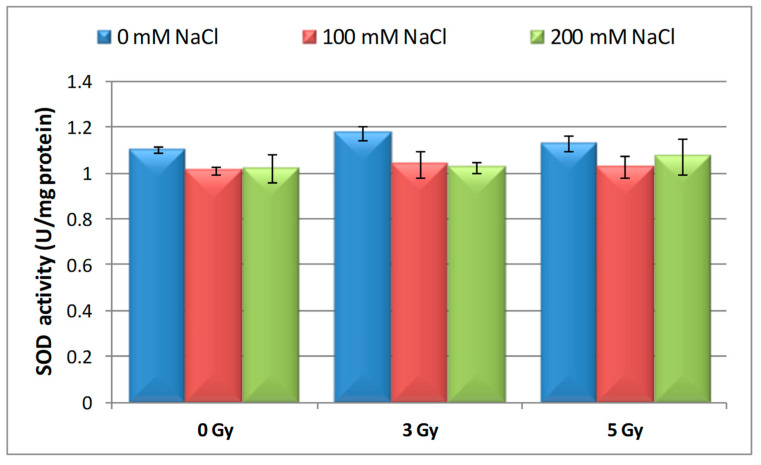
Effect of proton beam irradiation doses on the superoxide dismutase (SOD) activity in 16-days-old seedlings *Hordeum vulgare* as response to salt stress.

SOD activity, which is the first line of cell defense against ROS generated by saline and radiation treatments, had higher values only at 3 Gy and 5 Gy irradiated seedlings compared to the control, with an increase of only 6.24% and 2.43%, respectively. Meanwhile, when barley seedling germinated on 100 mM and 200 mM NaCl, the activity of SOD had relatively close values, being lower compared to the control (8.48% and 7.45%) (Figure 2). There was no major difference in response to SOD activity in 16-days-old barley seedlings irradiated with 3 and 5 Gy and grown under saline stress conditions (200 mM and 100 mM NaCl).

**Figure 3:**
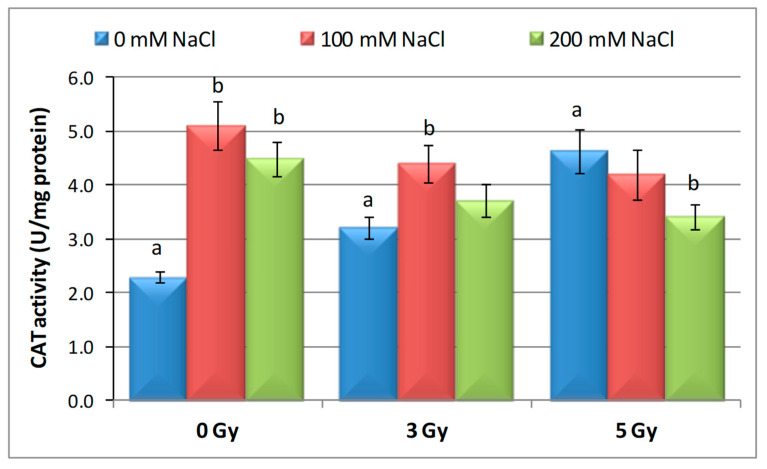
Effect of proton beam irradiation doses on the CAT activity in 16-days-old seedlings *Hordeum vulgare* as response to salt stress. Values are mean of three experiments ± SE; letters are designating the significant difference between samples and control group.

Out of the three antioxidant enzymes studied, CAT showed the highest enzymatic activity in response to alone actions of protons compared with the control (Figure 3). Single proton 3 Gy beam irradiation determined an increase in CAT activity by 11.61%, while in the case of 5 Gy irradiation the increase was 132%. On the other hand, there were no major differences in the CAT response to the two concentrations of NaCl (100 mM and 200 mM NaCl) applied alone but the increase was by 113.41% and 110.99% when compared to the control. In barley seeds irradiated with 3 Gy, which have germinated on 100 mM and 200 mM NaCl, CAT activity increased by 104% and 40%, respectively, compared to the control. In contrast, CAT activity showed a 40.12% and 38.62% increase compared to the control in 5 Gy-irradiated barley seeds and germinated on 100 mM and 200 mM NaCl.

**Figure 4:**
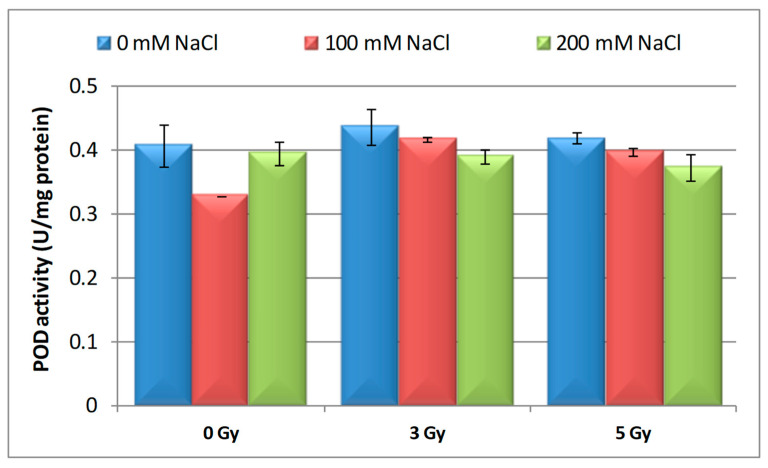
Effect of proton beam irradiation doses on the POD activity in 16-days-old seedlings *Hordeum vulgare* as response to salt stress.

When compared to the control, there was little increase in POD activity (7.38%, 2.75%, and 2.24%) after barley seeds irradiation with the two doses (3 and 5 Gy), as well as after 3 Gy irradiation of seedlings germinated on 100 mM NaCl (Figure 4, Table 3). It is interesting to note the fact that under saline conditions a decrease in POD activity (19.38% and 3.48%) was observed in barley seedlings germinated on 100 mM and 200 mM NaCl compared to the control. POD activity was influenced more intensely when 3 and 5 Gy irradiated barley seeds germinated in the presence of 200 mM than of 100 mM NaCl if we refer to the seeds irradiated only with the appropriate doses. Effect of proton beam radiation doses on antioxidant enzymes (SOD, CAT, and POD) activity investigated in this study on 16-days-old barley seedlings in response to the salt stress was heterogeneous.

The generation of reactive oxygen species (ROS) can be induced by diverse abiotic stresses, such as salt, drought, heat, or heavy metal stress. ROS is a collective term used both for free radicals, like hydroxyl radical (•OH), superoxide anion (•O_2_^−^), and alkoxy radicals (RO•) as well as non-radical (molecular) forms such as hydrogen peroxide (H_2_O_2_) and singlet oxygen (^1^O_2_) [58,59]. Salinity-mediated hyperosmotic and hyperionic stress causes supplementary stress in plants, well-known as oxidative stress. This phenomenon is due to the disruption of the balance between ROS generation and its decrease by various antioxidants [60].

In order to cope with different types of environmental stresses, plants have developed various strategies. Thus, in response to ROS overproduction, plant cells activate endogenous enzymatic defense mechanisms against oxidative stress [61]. To maintain the balance of ROS levels, plants synthesize various enzymatic and non-enzymatic antioxidants that equilibrate but do not eliminate ROS, because complete eradication of ROS means the loss of a second important messenger in intracellular signaling cascades [62]. The ROS excess can lead to irreparable metabolic dysfunction and even cell death by direct attack of photosynthetic pigments, membrane lipids, proteins, and nucleic acids [63].

Induction of the antioxidant defense system is an important mechanism of stress response after irradiation and salinity treatment. Thus, it has been shown that gamma rays can interact directly with different cellular components by crossing multiple levels to reach membranes, proteins, and nucleic acids [64]. Several studies have reported an intensification of antioxidant enzymes activity in plants in response to various ionizing radiation treatments [18,38,65].

Our research revealed that CAT had more intense activity than SOD, both in single and combined treatments applied to barley seeds and seedlings. These results are in accordance with those of Reference [65] on the irradiated and non-irradiated *Arabidopsis* sp. samples. The authors established that low-dose gamma irradiations induced a significant increase in the antioxidant enzymes POD, SOD, and CAT activity in *Arabidopsis* sp seedlings. SOD, which forms the first and most important line of antioxidant defense, catalyzes the rapid dismutation of •O_2_^−^ to produce H_2_O_2_. However, the H_2_O_2_ produced by SOD is toxic to plant cells and may be broken down into H_2_O and O_2_ by the action of CAT or POD [66]. Baek et al. [49] showed that the activity of some antioxidant enzymes, such as superoxide dismutase (SOD) and ascorbate peroxidase (APX), rose by increasing NaCl concentrations, and the irradiated groups had higher SOD and APX activity compared to the non-irradiated ones. The alleviation action was similar in both rice cultivars tested.

Antioxidant enzymes activity in seeds and young leaves of colored wheat seedling had diverse responses after acute (dose rate: 12.5 Gy/h, 37.5 Gy/h, and 62.5 Gy/h) and chronic (dose rate: 0.298 Gy/h, 0.893 Gy/h, and 1.488 Gy/h) exposure times (two weeks) [67]. The authors mention that POD activity in seeds was higher after chronic irradiation, and CAT activity was similar to that in control seeds, but lower after acute irradiation. SOD activity did not change significantly in the case of the two types of irradiation. The POD activity in seedlings showed a similar pattern in both applied treatments and CAT activity increased after chronic irradiation. Chronic irradiation induced higher SOD activity in colored wheat seedlings.

As far as our results are concerned, the differences observed in CAT and POD enzyme activities can be argued by the affinity of both enzymes for H_2_O_2_. Even though both enzymes are stimulated by H_2_O_2_ accumulation, CAT is activated at higher concentrations while POD is triggered at low H_2_O_2_ levels [68].

Zaka et al. [69] explained that after the application of low doses of radiation, the enzymatic antioxidants activity has increased due to increased regulation of the corresponding genes to provide cells resistance. At high doses, cellular oxidative stress plays a significant role in cell damage caused by ionizing radiation, and antioxidants can have a protective effect at these doses [70].

The treatment with proton beams reduced chlorophyll content in the soybean seedlings [56]. Moreover, the authors mention that the main antioxidant enzymes, SOD and POD, have similar pattern of activity in two soybean cultivars, with the effect being more intense at low doses (55 Gy).

### 3.4. Effect of Salinity and Proton Irradiation on MDA Content of Barley Seedling

MDA is the end product of lipid peroxidation and represents one of the most illustrative tests for assessing oxidative damage in plant tissues, being a major indicator of oxidative stress [71].

**Figure 5:**
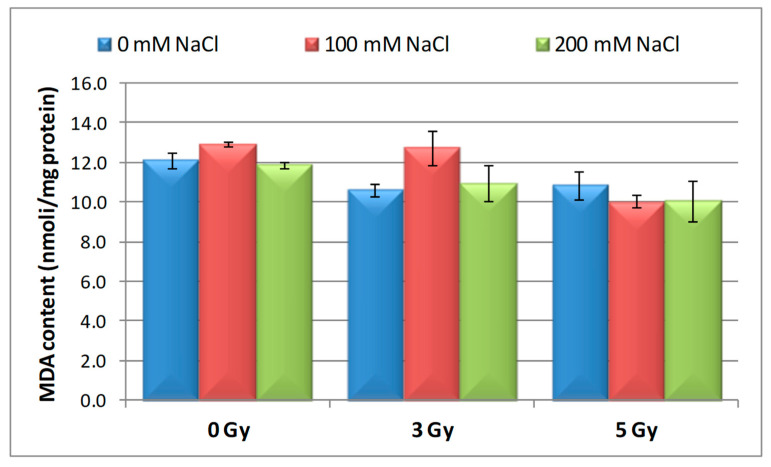
Effect of proton beam irradiation doses on the malondialdehyde (MDA) content in 16-days-old seedlings *Hordeum vulgare* as response to salt stress.

To see whether irradiation with proton beam could mitigate the oxidative stress induced by salinity in barley seedlings, MDA content was measured. As shown in Figure 5, MDA activity decreased both at 3 and 5 Gy irradiated barley seeds by a rate of 12.55% and 10.41%, compared to the control. Moreover, a gradual decrease in MDA content was observed with the increase of NaCl concentration in the single saline solution application. The highest MDA level (stimulation rate over 6%) was registered in seeds with 100 mM NaCl exposure. The seeds irradiated with 3 Gy and germinated in the presence of 100 mM NaCl revealed the increase of MDA level (12.74 nmol mg^−1^ protein), while 200 mM NaCl stress induced a decline (10.92 nmol mg^−1^ protein) of this enzyme activity, compared to the control (12.11 nmol mg^−1^ proteins). Both saline concentrations (100 mM and 200 mM) applied to seeds after irradiation with 5 Gy determined the inhibition of MDA content by 17% and 16%, respectively.

Our results showed that 5 Gy irradiation can effectively reduce the oxidative stress of 100 mM and 200 mM NaCl seedlings, indicated by lower levels of MDA in the irradiated material than in non-irradiated seedlings, as response to both saline stresses.

After proton beam pretreatment (3 and 5 Gy), the SOD activity in barley seedlings was antagonistic to the MDA content, the main role of SOD being as a scavenger for radicals superoxide, having as effect inactivation of CAT and POD activity [56].

Recent studies [72] have demonstrated that pretreatments with gamma irradiation caused a decrease of MDA activity in *Vicia sativa* L. Using high doses of irradiation (50–400 Gy, corresponding to 57 MeV), Reference [47] revealed an increase of MDA content in soybean plants irradiated with proton beam, without a direct dose-effect relationship.

Lipid peroxidation induced by free radicals can generally determine an increase in membrane permeability or a loss of its integrity, leading to a higher level of MDA [73]. Thus, the changes in MDA activity can serve as an index for determining the level of membrane permeability and integrity. Under conditions of salt stress, the cell membrane is damaged as a response to a high level of lipid peroxidation [74]. Although MDA typically increases when plants are subjected to salt stress, MDA level improved by up to 3–7% in rice seedling using 4 Gy gamma-irradiated seeds and germinated in presence of 40 mM NaCl [49].

Our results are similar to those of Song et al. [75] who, using resistant (ST-salt tolerance) and sensitive (WT-weak tolerance) mutant rice lines, found an increase in MDA level due to prolonged salt stress, with this augmentation being higher in the WT lines than in the ST plants.

### 3.5. Effect of Salinity and Proton Irradiation on Soluble Protein Content of Barley Seedling

**Figure 6:**
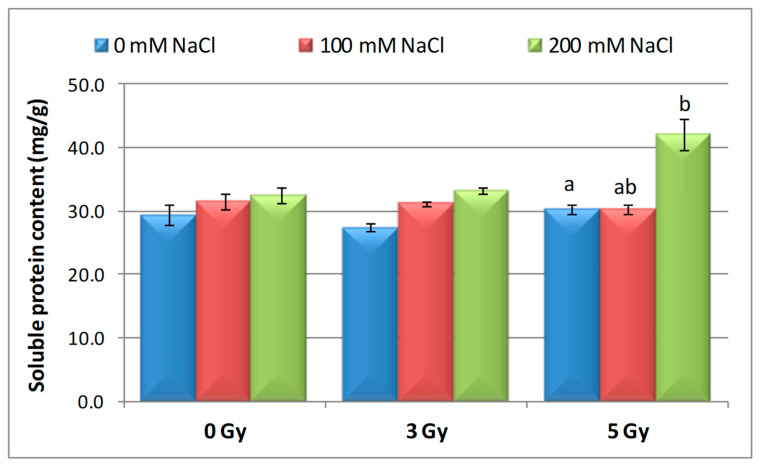
Effect of proton beam irradiation doses on the protein content in 16-days-old seedlings *Hordeum vulgare* as response to salt stress. Values are mean of three experiments ± SE; letters are designating the significant difference between samples and control group.

Different changes in soluble protein content of barley samples were observed in non-irradiated (0 Gy) and irradiated (3 and 5 Gy) seedling, with and without NaCl-associated treatment (100 mM and 200 mM). Thus, after irradiation with 3 Gy, the protein content was lower by 6.58% than the control, and the 5 Gy dose determined an insignificant increase in this parameter (by 2.98%), as shown in Figure 6. Our results proved that the soluble protein content rose with increasing NaCl concentrations. Seeds irradiated with both 3 Gy and 5 Gy showed an increase in protein content in the presence of 200 mM compared to 100 mM NaCl. Interesting enough, when the 5 Gy irradiated seeds were germinated in the presence of 200 mM NaCl, the soluble protein content was 36.53% higher than control.

The increase in the content of soluble protein is one of the protective mechanisms of the plants against gamma irradiation damages, which correlates with increasing doses [76]. Thus, Štajner et al. [77] established that gamma irradiation (1–10 kGy) of soybeans has induced insignificant changes in the soluble protein content. Similar results were obtained by Byun et al. [78] who mentioned that gamma irradiation, up to 10 kGy, did not induce significant reduction of minerals, nitrogenous constituents, sugars, and proteins content. On the other hand, Hanafy and Akladious [40] reported that high dose of gamma radiation (400 Gy) led to a significant decrease in the total protein contents in fenugreek plants as compared to untreated control plants.

### 3.6. Effect of Salinity and Proton Irradiation on Content of Total Polyphenol and Flavonoids in Barley Seedlings

Apart from antioxidant enzymes, small molecular weight and non-enzymatic antioxidants are also involved in the protection of the intracellular components against the ROS.

**Figure 7:**
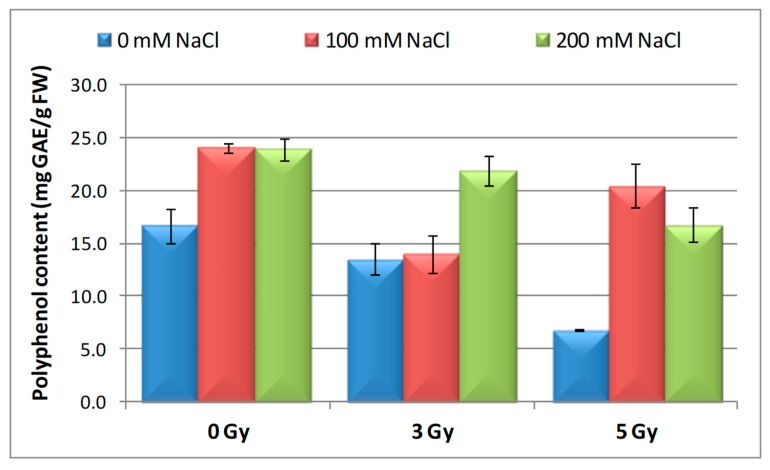
Effect of proton beam irradiation doses on the polyphenol content in 16-days-old seedlings *Hordeum vulgare* as response to salt stress.

In our research, proton beam irradiation (3 and 5 Gy) applied alone led to a marked decline in polyphenols content of barley seedlings (Figure 7) compared to the control (18% and 59%, respectively). The decrease was much more significant at 5 Gy. The highest increase of polyphenols level (44.23 and 43.18%, respectively) was observed when barley seeds germinated only in the presence of NaCl concentrations (100 and 200 mM). The application of 100 mM NaCl on the proton beam irradiated 3 Gy seeds showed a decrease (by 16%) in the polyphenol content while the treatment with 200 mM NaCl determined an increase by 31%, compared to the control. We mention that the situation was exactly the opposite for the seeds irradiated with 5 Gy and grown under 100 mM and 200 mM NaCl conditions. Due to their hydroxyl groups with scavenging ability, phenolics are important constituents and may hence contribute directly to the antioxidant action [79].

Low doses of radiation stimulate the accumulation of antioxidants, and synthesis can be induced by an increased gene expression and intensification of monomers degradation of some polyphenols. The high-dose radiation reduces the content of non-enzymatic and enzymatic antioxidants because the plant loses its ability to defeat the oxidative damage [53,80].

**Figure 8:**
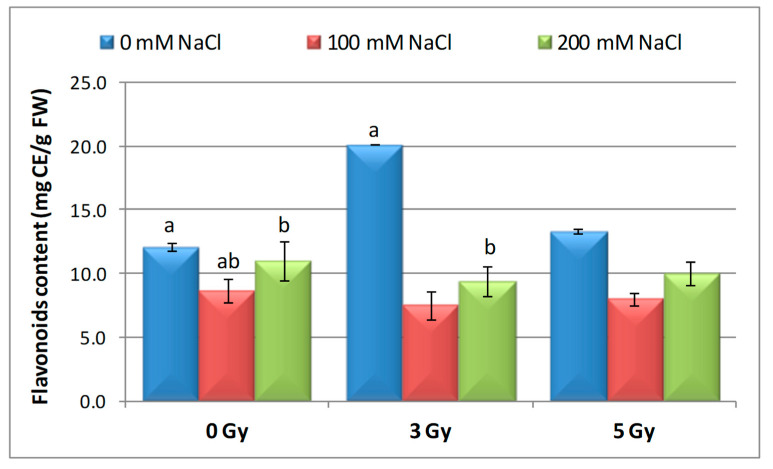
Effect of proton beam irradiation doses on the flavonoids content in 16-days-old seedlings *Hordeum vulgare* as a response to salt stress. Values are mean of three experiments ± SE; letters are designating the significant difference between samples and control group.

Regarding the flavonoids content (Figure 8), the results revealed increasing values (by 66% and by 10%, respectively) only in the case of seeds irradiated with proton beam (3 Gy and 5 Gy), compared with the control. Both saline treatments showed a decrease in the level of flavonoids in barley seedlings, with 28.43% in the case of 100 mM NaCl and 9.04% in 200 mM NaCl lower than the control. Moreover, when the seeds irradiated with the proton beam were germinated in saline conditions, the decrease in flavonoids content was more intense at 100 mM NaCl, compared to the control, with 37.97% and 33.91% in the case of irradiation with 3 Gy and 5 Gy, respectively. This reduction was lower at a saline stress of 200 mM registering a decrease rate of 22.33% and 17.29% (for 3 Gy and 5 Gy). Moreover, when the proton beam-irradiated seeds were germinated on saline conditions, it maintained the decrease of this parameter higher at 100 mM NaCl (5.26% and 3.18% for 3 Gy and 5 Gy) and lower at 200 mM NaCl (13.48% and 51.91% for 3 Gy and 5 Gy).

Flavonoids represent one of the secondary metabolites with significant antioxidant and chelating properties, which are present in plants and have a significant role in the reduction of damage induced by different types of irradiation.

Exposure of fenugreek seeds to gamma radiation (25, 50, 100, 200, and 400 Gy) induced an increase in the phenolic and flavonoids content, especially at 100 Gy, both in M1 and M2 generation. Instead, the high dose of 400 Gy decreased all physiological and biochemical parameters analyzed [40]. Other studies [81] signaled that gamma irradiation (2, 4, 8, 16, 32, and 64 k-rad) associated with bio-fertilizer treatments have increased the total level of flavonoids in dill seedlings, with the highest content recorded at 64 k-rad. Furthermore, the flavonoids accumulation depends on the analyzed parts of the plant (umbels > vegetative plant > flowering plant > leaves + stems in the flowering stage).

## 4. Conclusions

The impact of synergistic exposure to proton beam irradiation of barley seeds and salinity stress was discussed in relation to the potential of enzymatic and non-enzymatic antioxidants and soluble protein content, as well as assimilatory pigment level.

The 3 Gy proton beam irradiation resulted in lower assimilatory pigments content compared to the control, even if the germination occurred in 100 or 200 mM NaCl conditions. Out of all the antioxidant enzymes that were part of the study (SOD, CAT, and POD) only CAT activity increased in irradiated seeds germinated under salinity conditions, which indicates the activation of antioxidant defense. The MDA content of seeds germinated on 200 mM NaCl stress dropped as the irradiation doses increased. The 3 and 5 Gy proton beam pretreatment of barley seeds, associated with 200 mM NaCl exposure, revealed a high soluble protein content in the 16-days-old seedlings. When compared to the control, the total polyphenol amount was lower only when the seeds germination was performed in the presence of 100 mM NaCl, while the flavonoids content was inhibited regardless of the applied salinity level. Based on the growth rate, the study suggests that 3 Gy proton beam irradiation of barley seeds can alleviate the harmful action of 100 mM NaCl stress, as proved by a 1.95% increase seedlings growth rate as compared to the control.

## Figures and Tables

**Table 1:** The effect of proton beam irradiation on 16-days-old *Hordeum vulgare* seedlings length in presence of salt stress. Means values (± SE) of 10 replicates.

Samples	Means Values (cm)	P<	%	−/+ Rate (%)
Control	14.50 ± 0.90			
3 Gy	14.30 ± 0.78	NS	98.62	−1.38
5 Gy	13.76 ± 0.78	NS	94.90	−5.10
100 mM NaCl	15.36 ± 0.99	NS	105.93	5.93
200 mM NaCl	6.00 ± 0.35	*p* < 0.001	41.38	−58.62
3 Gy + 100 mM NaCl	14.78 ± 0.49	NS	101.93	1.93
3 Gy + 200 mM NaCl	3.93 ± 0.27	*p* < 0.001	27.10	−72.90
5 Gy + 100 mM NaCl	13.89 ± 0.59	NS	95.79	−4.21
5 Gy + 200 mM NaCl	3.78 ± 0.33	*p* < 0.001	26.07	−73.93

**Table 2:** Modulation of the photosynthetic pigments content in 16-days-old seedlings *Hordeum vulgare* as response to salt stress and proton beam irradiation.

Sample	Chlorophyll *a* (mg g^−1^ FW)	Chlorophyll *b* (mg g^−1^ FW)	Carotenoids (mg g^−1^ FW)
Control	0.622 ± 0.026 ^a^	0.160 ± 0.008 ^a^	0.143 ± 0.005 ^a^
3 Gy	0.482 ± 0.064	0.129 ± 0.018	0.095 ± 0.017
5 Gy	0.649 ± 0.132	0.165 ± 0.029	0.130 ± 0.043
100 mM NaCl	0.433 ± 0.079	0.126 ± 0.015	0.111 ± 0.019
200 mM NaCl	0.345 ± 0.039 ^b^	0.101 ± 0.008 ^b^	0.074 ± 0.009 ^b^
3 Gy + 100 mM NaCl	0.267 ± 0.013 ^b^	0.072 ± 0.015 ^b^	0.061 ± 0.013
3 Gy + 200 mM NaCl	0.371 ± 0.002 ^b^	0.103 ± 0.012	0.074 ± 0.006 ^b^
5 Gy + 100 mM NaCl	0.434 ± 0.092	0.239 ± 0.028	0.117 ± 0.014
5 Gy + 200 mM NaCl	0.570 ± 0.045	0.173 ± 0.008	0.108 ± 0.009

Values are mean of three experiments ± SE; letters are designating the significant difference between samples and control group.

**Table 3:** Modulation of the main biochemical indicators after proton beam irradiation in 16-days-old seedlings *Hordeum vulgare* as response to salt stress.

Samples	SOD Activity	−/+ Rate (%)	CAT Activity	−/+ Rate (%)	POD Activity	−/+ Rate (%)	MDA Content	−/+ Rate (%)	Flavonoid Content	−/+ Rate (%)	Polyphenol Content	−/+ Rate (%)	Soluble Protein Content	−/+ Rate (%)
Control	1.10	0.00	2.30 ^a^	0.00	0.40	0.00	12.11	0.00	12.09 ^a^	0.00	16.67	0.00	29.34	0.00
3 Gy	1.17	6.36	2.56	11.30	0.43	7.50	10.60	−12.47	20.11	66.34	13.52	−18.90	27.41	−6.58
5 Gy	1.13	2.73	5.35	132.61	0.41	2.50	10.85	−10.40	13.32	10.17	6.82	−59.09	30.22 ^a^	3.00
100 mM NaCl	1.00	−9.09	4.90 ^b^	113.04	0.32	−20.00	12.93	6.77	8.65 ^ab^	−28.45	24.04	44.21	31.56	7.57
200 mM NaCl	1.02	−7.27	4.85 ^b^	110.87	0.39	−2.50	11.89	−1.82	11.00 ^b^	−9.02	23.87	43.19	32.57	11.01
3 Gy + 100 mM NaCl	1.03	−6.36	4.69 ^b^	103.91	0.41	2.50	12.74	5.20	7.50	−37.97	13.98	−16.14	30.89	5.28
3 Gy + 200 mM NaCl	1.02	−7.27	3.22	40.00	0.38	−5.00	10.92	−9.83	9.39 ^b^	−22.33	21.87	31.19	33.30	13.50
5 Gy + 100 mM NaCl	1.02	−7.27	3.23	40.43	0.39	−2.50	10.04	−17.09	7.99	−33.91	20.45	22.68	30.28 ^a b^	3.20
5 Gy + 200 mM NaCl	1.07	−2.73	3.18 ^b^	38.26	0.37	−7.50	10.08	−16.76	10.00	−17.29	16.75	0.48	40.06 ^b^	36.53

Values are mean of three experiments ± SE; letters are designating the significant difference between samples and control group.
